# Immunotherapy in the Treatment of Head and Neck Squamous Cell Carcinoma: A Review of Current Evidence, Emerging Strategies and Future Directions

**DOI:** 10.3390/biology15201851

**Published:** 2026-10-15

**Authors:** Bayle Smith-Salzberg, Lucas Resende Salgado, Catherine Yu, Danielle Arons, Nicholas Murphy, Anthony D. Nehlsen, Kristin Hsieh, Kunal K. Sindhu

**Affiliations:** 1 College of Medicine, State University of New York (SUNY) Downstate Health Sciences University, Brooklyn, NY 11203, USA; 2 Department of Radiation Oncology, Icahn School of Medicine at Mount Sinai, New York, NY 10029, USA; 3 New York Proton Center, New York, NY 10035, USA

**Keywords:** head and neck cancer, checkpoint inhibitors, immunotherapy, chimeric antigen receptor T-cell therapy (CAR-T cell)

## Abstract

*Introduction:* Head and neck squamous cell carcinomas (HNSCCs) are among the most common cancers worldwide and continue to carry substantial morbidity and mortality despite advances in surgery, radiation therapy (RT), and systemic therapy. Treatment varies according to disease stage, clinical setting, and prior therapy. The immunologically active tumor microenvironment of HNSCC and expression of immune-regulatory pathways have made immunotherapy an important area of therapeutic development. *Methods:* This is a narrative review of the established and emerging immunotherapeutic strategies for HNSCC, their clinical applications, mechanisms of resistance, and future directions. We used relevant search terms in PubMed to examine the available literature. *Discussion and Conclusions:* We discuss several immunotherapeutic agents, including PD-1, PD-L1 and CTLA-4 inhibitors, as well as emerging immune checkpoint targets such as LAG-3, TIGIT, TIM-3 and VISTA. We also consider the importance of HPV status in prognosis and treatment response. The role of immunotherapy is considered across disease stages and treatment settings, with particular emphasis on recurrent or metastatic HNSCC and locally advanced HNSCC in the perioperative setting, for which PD-1 inhibitors have demonstrated the clearest clinical benefit and have become an important component of standard therapy. We also review the expanding role of neoadjuvant and adjuvant immunotherapy, as well as combinations with RT, for which efficacy remains under investigation or has been inconsistent across clinical trials. In addition, we examine mechanisms of primary and acquired resistance related to tumor biology and molecular mutations. Finally, we highlight emerging therapeutic approaches, including chimeric antigen receptor T-cell therapy, therapeutic cancer vaccines, and combinations of immunotherapy with proton beam therapy. Together, these developments illustrate the rapidly expanding role of immunotherapy across HNSCC treatment and identify areas in which further investigation may expand therapeutic options and improve outcomes.

## Simple Summary

This review examines the current and emerging use of immunotherapy in the treatment of head and neck squamous cell carcinoma. It discusses potential and currently clinically relevant immune targets, including PD-1, PD-L1, CTLA-4, LAG-3, TIGIT, TIM-3, and VISTA, and the importance of HPV status on prognosis and treatment response. It proceeds to explore different stages of disease and treatment settings where immunotherapy has been explored or already incorporated into the standard-of-care treatment: recurrent, metastatic, or locally advanced disease, and neoadjuvant, adjuvant, perioperative, or concurrent treatment with radiotherapy. The mechanisms of resistance to immunotherapy are then described. Finally, other advanced immunotherapies and combination therapies are reviewed: adoptive T-cell therapy, therapeutic vaccines, and immunotherapy combined with proton beam therapy.

## 1. Introduction

### 1.1. Background

Head and neck cancer (HNC) is the seventh most prevalent cancer worldwide and is increasing in incidence [1,2]. Approximately 90% of cases of HNC are squamous cell carcinomas (HNSCCs), which are derived from the mucosal epithelial lining of the larynx, pharynx, or oral cavity [1,3]. Alcohol use, tobacco exposure, and human papillomavirus (HPV) infection are major risk factors for HNSCC, while Epstein–Barr virus (EBV) is associated with nasopharyngeal carcinoma [4,5,6,7]. The stage at which HNSCC is diagnosed strongly predicts overall survival (OS); while the five-year survival rate for stage I-II disease is around 70–90%, it is 40% for locoregionally advanced disease [8]. In addition to early stage disease, a positive HPV status indicates a more favorable prognosis [9]. Unfortunately, most cases of HNSCC are detected at the locoregionally advanced stage (stages III or IV), and, thus, the overall five-year survival of HNSCC is around 50%, a figure that has not seen much improvement in recent years [1,10,11].

Treatment of HNSCC is multimodal and often involves a combination of radiation therapy (RT), chemotherapy, and surgery. HNSCC is susceptible to RT and cisplatin-based therapy, and, therefore, chemoradiotherapy has been a core treatment modality employed [8]. Surgery can also be effective, but the tumor’s location and extent of invasion in the head and neck region must be carefully considered when weighing the therapeutic benefit of surgery against potentially detrimental functional and quality of life outcomes [8]. Until recently, treatment progress has been incremental, characterized by combining established modalities and improving RT techniques. However, over the past decade, the advent of immunotherapy has demonstrated promise in advancing HNSCC treatment.

### 1.2. Rationale and Knowledge Gap

HNSCCs are considered a particularly good target for immunotherapy among solid tumor malignancies. In general, tumors evade destruction by immune cells by activating inhibitory checkpoints and thriving in a low-oxygen, cytokine-rich microenvironment that dampens immune surveillance [12]. HNSCCs produce a large number of neoantigens, new antigens on the surface of cancer cells, due to a high somatic mutation rate [13]. These neoantigens may serve as targets for T-cell recognition, thereby increasing tumor immunogenicity. Thus, compared to other cancers, HNSCCs are greatly infiltrated by regulatory T cells (Tregs) and natural killer (NK) cells, according to a transcriptome analysis [14]. Increased immunological activity, especially involving CD8^+^ T cells and CD56^dim^ NK cells, in the HNSCC tumor microenvironment (TME) is associated with improved survival [14]. Since HNSCCs contain numerous immunologically active antigens and a stronger immune response has been associated with improved survival, it follows that targeted treatments that activate the host immune system could improve survival [14]. RT and chemotherapy both enhance the immune response and are associated with increased CD8^+^ and programmed cell death protein-1 (PD-1)-expressing T-cell activity and Treg suppression, making the tumors more susceptible to immunotherapy against these pathways [15]. Therefore, recent advances in HNSCC treatment have focused on immunotherapy and combining immunotherapy with RT and chemotherapy.

### 1.3. Objective

Immunotherapy options for HNSCC are currently built on immune checkpoint inhibitors (ICIs). In this review, we discuss immune targets for HNSCC. We then investigate the current use of ICIs in HNSCC. Finally, we explore emerging and future applications of immunotherapy in patients with HNSCC, which include bispecific antibodies, chimeric antigen receptor T-cell (CAR-T cell) therapy, therapeutic cancer vaccines, and combinations of immunotherapy with proton beam therapy.

## 2. Methods

This is a narrative review. A targeted literature search of PubMed was conducted from 1 February to 13 May 2026. The literature was subsequently updated during manuscript revision through September 2026. Search terms included “immunotherapy,” “head and neck cancer,” “PD-L1,” “PD-1,” “CAR-T cell therapy,” “therapeutic vaccines,” and “chemoradiotherapy.” These and related terms were used alone and in combination, including “PD-L1 in head and neck cancer,” “immune checkpoint inhibitors,” and “pembrolizumab.” There was no publication date limit to include foundational mechanistic studies and historically important work. Abstracts of identified articles were reviewed for relevance to modern practice with an emphasis on articles published from 2016 to 2026. Articles were selected for full-text review based on relevance to clinical practice, mechanistic understanding, or novelty. Included articles were clinical trials, observational studies, preclinical studies, or reviews.

## 3. Immune Targets

### 3.1. PD-1/PD-L1

The PD-1 receptor and its ligands, programmed death ligand-1 and ligand-2 (PD-L1 and PD-L2), comprise a key immune-checkpoint pathway exploited by many cancers. PD-1 is expressed primarily on activated T lymphocytes (CD4^+^ and CD8^+^), and can also be found on B cells, NK cells, and other myeloid antigen-presenting cells (APCs). With an intact immune system, T cells activate cell death signals when their T-cell receptor binds to certain peptide-major histocompatibility complexes (MHCs) on foreign cells [16]. Tumor cells that express PD-L1 bind to PD-1 to modulate T-cell signaling and cytotoxic activity, allowing tumor cells to escape immune-mediated elimination [16,17]. The PD-1/PD-L1 interaction triggers an activation cascade involving the Src family of kinases phosphorylating PD-1’s intracellular immunoreceptor tyrosine-based inhibitory motif (ITIM) and immunoreceptor tyrosine-based switch motif (ITSM) and recruiting Src homology region 2 domain-containing phosphatases 1 and 2 (SHP-1 and SHP-2). SHP-2 can then inhibit CD28-mediated costimulatory signaling via the dephosphorylation of CD28 [16,18,19,20,21]. Additionally, PD-L1 can separately interact with CD80 to inhibit T-cell activation [22,23,24]. Experimental studies have identified tumor-cell-intrinsic PD-L1 signaling that protects cancer cells against interferon-γ (IFN-γ)-mediated cytotoxicity independent of PD-1 engagement [25]. Conversely, eliminating PD-1 increases T-cell responsiveness, which causes an overactive immune response; thus, gene-deficient animal models may develop autoimmune disease or diabetes. Similarly, anti-PD-L1 agents in HNSCC immunotherapy have been associated with the development of autoimmune endocrinopathies [17].

Increased expression of PD-L1 in cancer cells occurs through several different mechanisms. Amplifications, translocations, and mutations in the IFN-γ signaling pathway, including to Janus kinase 2 (JAK2), and changes to microRNAs can all lead to increased expression of PD-L1 [17]. While IFN-γ is the main inducer of PD-L1, lipopolysaccharides, by way of toll-like receptor 4, and nuclear factor kappa-light-chain-enhancer of activated B cells (NF-κB), in addition to hypoxia-inducible factors (HIFs), also regulate PD-L1 expression [17]. In HNSCC specifically, overexpression of miRNA-217 has been shown to suppress PD-L1 protein expression in preclinical models [26,27].

Since both the interaction of PD-1/PD-L1 and the independent presence of PD-L1 protect against cell death, therapeutic strategies may target either or both to promote cell death in tumor cells. Over the past decade, many clinical trials have investigated the potential of PD-1 or PD-L1 inhibitors in the treatment of HNSCC. Nivolumab, an anti-PD-1 antibody, has been tested in the neoadjuvant, recurrent/metastatic (R/M), and postoperative settings with promising results [28,29,30,31]. Pembrolizumab is another established anti-PD-1 antibody that has been used in the treatment of many cancers. In HNSCC, it has been studied in the neoadjuvant, locally advanced, perioperative, and R/M settings, and concurrently with chemoradiotherapy [32,33,34,35,36]. Additionally, the PD-1 inhibitors toripalimab, tislelizumab, and camrelizumab have demonstrated encouraging results in the R/M setting [37,38,39,40,41,42]. On the other hand, some anti-PD-L1 antibodies, including avelumab, atezolizumab, and durvalumab, have failed to show improvement over standard of care [43,44,45,46,47]. Overall, certain anti-PD-1 agents have demonstrated clear efficacy in R/M HNSCC, and ongoing studies have shown mixed results in locally advanced disease, with recent positive phase III results in the perioperative setting and with many studies still ongoing.

### 3.2. CTLA-4

Cytotoxic T Lymphocyte Antigen 4 (CTLA-4) is historically significant as the first immune checkpoint receptor targeted in clinical practice. CTLA-4 is constitutively expressed at high levels on regulatory T cells (Tregs) and is upregulated on conventional T cells following T-cell activation [48]. CTLA-4 is an inhibitory immune checkpoint receptor that is expressed on T cells and has a higher binding affinity for CD80 (B7-1) and CD86 (B7-2), which are expressed primarily on APCs, than does the costimulatory receptor CD28 [48]. Binding of CD80/CD86 on APCs to CD28 provides a costimulatory signal that promotes T-cell activation, whereas CTLA-4 competes for these ligands and limits CD28-mediated costimulation [48]. CTLA-4 can also remove CD80 and CD86 from the surface of APCs through trans-endocytosis, which reduces their availability for CD28 binding and thus contributes to immune suppression [49].

Antibodies targeting CTLA-4 interfere with this inhibitory pathway and can therefore enhance antitumor T-cell activity [48,50]. Ipilimumab and tremelimumab are anti–CTLA-4 antibodies that are FDA-approved [51,52]. In HNSCC, neoadjuvant nivolumab plus ipilimumab demonstrated encouraging pathological responses in the phase Ib/IIa IMCISION trial [53]. However, most of the trials evaluating the role of CTLA-4 in HNSCC, particularly in the metastatic setting and in combination with anti-PD-1 antibodies, have failed to show an improvement in OS [54,55,56].

### 3.3. LAG-3

Lymphocyte activation gene-3 (LAG-3) is an inhibitory immune checkpoint expressed on activated T cells and plays a key role in the suppressive function of Tregs [57]. Preclinical studies have demonstrated that co-expression of LAG-3 and PD-1 on the surface of tumor infiltrating lymphocytes (TILs) is associated with downregulation of T cells, which may allow tumors to evade the immune response [58]. In HNSCC, LAG-3 is expressed on the surface of Tregs, providing a biological rationale for investigating LAG-3 blockade as a therapeutic strategy [59]. Primary ligands for LAG-3 are the MHC class II molecules, specifically stable peptide-MHC class II complexes and fibrinogen-like protein 1, suggesting possible targets for antibodies [60,61]. However, the evidence for LAG-3 inhibition in patients with HNSCC is limited to early phase trials, and, presently, there are no FDA-approved LAG-3 inhibitors for HNSCC [62].

### 3.4. TIGIT

T-cell immunoreceptor with immunoglobulin and immunoreceptor tyrosine-based inhibitory motif domains (TIGIT), a member of the poliovirus receptor-related-like protein family, is an inhibitory immune checkpoint that regulates antitumor immune responses [63]. TIGIT is expressed on immune cells, including activated CD4^+^ T helper cells, CD8^+^ cytotoxic T cells, γδ T cells, and Tregs [63,64,65]. Similarly to LAG-3, TILs have been shown to overexpress TIGIT relative to the peripheral T-cells, although these findings are not specific to HNSCC [66].

TIGIT signaling can inhibit NK cell and effector T lymphocyte function and can enhance Treg cell suppressive activity [67,68,69]. Engagement of TIGIT by CD155 suppresses NK- and T-cell function and shifts the TME towards an immune-regulatory state by prompting the release of anti-inflammatory IL-10 while downregulating proinflammatory cytokines, thereby inhibiting the antitumor immune response [63,70].

CD155 overexpression has been associated with poor overall survival in HNSCC [71]. Additionally, in preclinical HNSCC models, anti-PD-L1 treatment alone upregulates CD155 on myeloid-derived suppressor cells, while anti-TIGIT treatment upregulates PD-L1 on myeloid-derived suppressor cells. These preclinical findings suggest a possible compensatory mechanism between the TIGIT/CD155 and PD-1/PD-L1 pathways that can provide a means of resistance to anti-PD-L1 or anti-TIGIT treatments as monotherapies, but also present a potential double blockade target [72]. In contrast to these preclinical findings, clinical data supporting TIGIT/CD155 blockade remain limited with most evidence arising from early-phase studies combining anti-TIGIT agents with RT [44].

### 3.5. TIM-3

T-cell immunoglobulin mucin-3 (TIM-3) is expressed on CD4+ Th1, Tregs, dendritic cells, monocytes, mast cells, NK cells and TILs [73,74]. Galectin-9 is a well-characterized ligand for TIM-3, and when bound, it promotes the apoptosis of Th1 cells, downregulating CD8+ T cells, inducing myeloid-derived suppressor cell propagation, and consequently slowing the immune response [75].

TIM-3 is significantly upregulated on TILs in HNSCC and is associated with lymph node metastasis, recurrent disease, and prior treatment with RT or chemotherapy [76]. In HNSCC models and patient samples, PD-1 and TIM-3 co-expression marks a more exhausted T-cell phenotype, with the highest PD-1 levels found on TILs co-expressing TIM-3 [77]. Galectin-9/TIM-3 signaling has also been shown to inhibit NK cell cytotoxicity [78]. Preclinical HNSCC studies have shown increased TIM-3 expression following PD-1 blockade, suggesting that TIM-3 upregulation may contribute to adaptive resistance to PD-1 inhibition [77]. Based on these mechanistic and preclinical observations, combined inhibition of TIM-3, PD-1, and LAG-3 is a potential target of clinical investigation in HNSCC. However, no anti-TIM-3 therapies have been approved for HNSCC and clinical efficacy has not been established [62].

### 3.6. VISTA

V-domain Ig suppressor of T-cell activation (VISTA) is an Ig domain-containing immune checkpoint molecule, which suppresses T-cell-mediated responses [79]. VISTA is expressed on hematopoietic cells and myeloid cells, including neutrophils, monocytes, macrophages and dendritic cells [80]. In human oral squamous cell carcinoma specimens, VISTA expression has been associated with PD-L1, CTLA-4, and myeloid-derived suppressor cell markers CD11b and CD33 [81]. These observational findings provide a biological rationale for investigating VISTA as an immune checkpoint target, but do not establish the clinical efficacy of anti-VISTA therapy in HNSCC.

**Figure 1:**
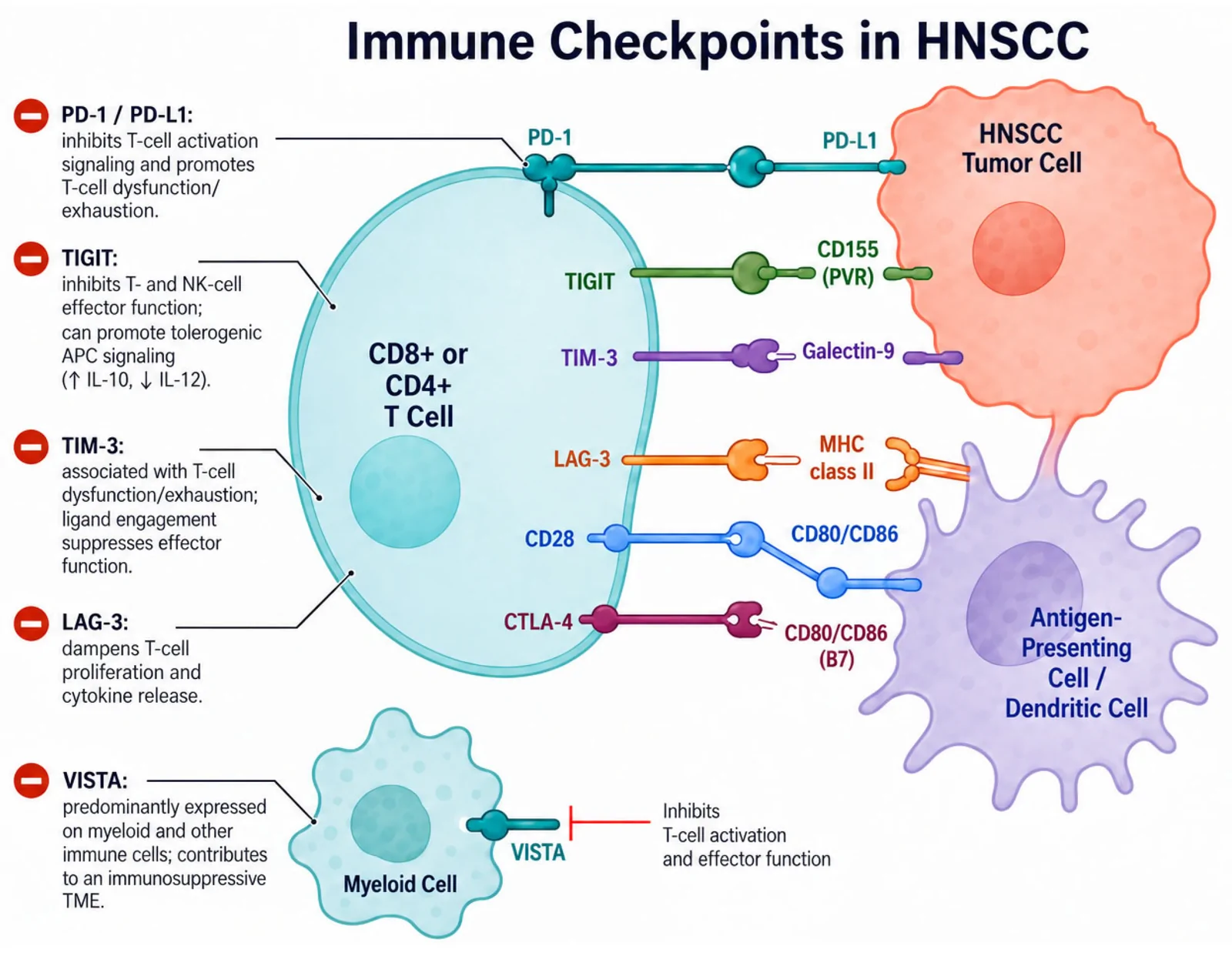
Generated using Claude Fable 5 (Anthropic) and ChatGPT GPT-5.6 Sol (OpenAI) based on author-provided content and instructions. The authors reviewed and edited the figure for accuracy. Schematic of immune checkpoint interactions among a CD8+ or CD4+ T cell, an HNSCC tumor cell, and an antigen-presenting/dendritic cell. PD-1/PD-L1, TIGIT/CD155, TIM-3/Galectin-9, LAG-3/MHC class II, CTLA-4/CD80/CD86, and VISTA-mediated signaling are shown as inhibitory pathways that suppress T-cell activation, while CD28 binding to CD80/CD86 provides a costimulatory signal to promote T-cell activation. Among these pathways, PD-1 blockade is an established therapeutic strategy in HNSCC, while targeting CTLA-4, TIGIT, TIM-3, LAG-3, and VISTA remains investigational in HNSCC. Abbreviations: TCR—T-cell receptor; CD—cluster of differentiation; B7—B7 family costimulatory ligands (CD80/CD86); PVR—poliovirus receptor (CD155); IL-10—interleukin-10; IFN-γ—interferon gamma; TLR—toll-like receptor.

The above targets are illustrated in Figure 1, below.

### 3.7. HPV Status

HPV status is an important biological and prognostic consideration in HNSCC and may also influence responses to immunotherapy. HPV-positive tumors express virus-derived antigens and generally demonstrate a more inflamed, or “hot,” TME characterized by greater immune cell infiltration and T-cell activation compared to HPV-negative tumors [82,83]. Typically, tumors with “hot” TMEs are associated with better treatment responses and improved outcomes when treated with immunotherapy [82]. HPV positivity has also been associated with a better prognosis in oropharyngeal cancer [84]. In a pooled analysis of patients with HPV-positive and -negative HNSCC treated with PD-1/PD-L1 inhibitors, those with HPV-positive disease showed improved overall survival and objective response rates compared to patients with HPV-negative disease [85]. However, HPV status has not consistently predicted benefit from ICIs across studies [82]. A meta-analysis of seven randomized controlled trials in locally advanced HNSCC found no significant difference in pooled time-to-event outcomes (event-free survival, progression-free survival, and disease-free survival) with ICI-based therapy compared to standard treatment in either HPV/p16-positive or -negative subgroups [86]. Another meta-analysis found a numerically higher response rate and longer overall survival among HPV-positive patients treated with immunotherapy, although the difference in response rate was not statistically significant [87]. Therefore, although some studies suggest improved outcomes with ICI-based treatment in HPV-positive disease, HPV status has not consistently emerged as an independent predictive biomarker of ICI benefit. Still, HPV-positive tumors present a distinct opportunity to target viral antigens such as E6 and E7 through therapeutic vaccines (see Section 6.3) [88].

## 4. Immunotherapy Use in Head and Neck Cancers

### 4.1. Recurrent and Metastatic Settings

R/M disease is common among patients with HNSCC, with more than 65% presenting with or developing R/M disease, including 10% who present with metastatic disease at diagnosis [89]. If the disease recurs, it is typically within 3 years of treatment completion. Despite treatment, patients with R/M HNSCC have a 5-year survival rate of <20%, with a median survival of 12–15 months [11].

Current guidelines for the treatment of R/M disease depend on previous therapies, resectability, extent of disease spread, and the availability of clinical trials. When resectable, surgery is often the next step, with consideration of adjuvant RT, chemotherapy, and/or immunotherapy depending on pathological risk factors. Unresectable cases may receive curative intent RT, which can include concurrent platinum-based chemotherapy and potentially the re-irradiation of previously irradiated sites. Based on recent trials, the first-line treatment for R/M HNSCC with no surgical or RT options now includes both chemotherapy and immunotherapy options [90].

In the early 2000s, systemic therapy for R/M HNSCC consisted only of chemotherapies [89]. A 2008 study established the benefit of adding cetuximab, an epidermal growth factor receptor (EGFR) inhibitor, to chemotherapy, which prolonged the median OS and progression free survival (PFS) compared to chemotherapy alone [91]. This was known as the EXTREME regimen [89,92].

Building on the success of an anti-EGFR antibody, immunotherapy agents targeting the PD-1 pathway were studied, and pembrolizumab and nivolumab were approved by the FDA in 2016 for R/M HNSCC [89]. These early trials examined their use as subsequent-line therapy for platinum-refractory disease. The phase III CheckMate 141 trial demonstrated that nivolumab significantly improved median OS over investigator’s choice systemic therapy [28]. For platinum-refractory R/M HNSCC, the KEYNOTE-012 trial, a phase Ib trial of pembrolizumab, demonstrated the drug’s tolerability and clinically meaningful response rate with potential durability [32]. These results allowed for the FDA’s accelerated approval of pembrolizumab for platinum-refractory R/M HNSCC [8]. Subsequently, KEYNOTE-040 showed improved OS in the R/M setting with pembrolizumab compared to the other established therapies: methotrexate, docetaxel, or cetuximab [11,33].

Immunotherapy was established as first-line treatment for R/M HNSCC in 2019 by the KEYNOTE-048 trial [89]. In this phase III study of patients with untreated, locally incurable R/M HNSCC, patients received one of three treatments: pembrolizumab alone, pembrolizumab in combination with chemotherapy (a platinum agent and 5-fluorouracil), or the EXTREME regimen (cetuximab with chemotherapy) [34]. Combined positive score (CPS) for PD-L1 expression was ≥20 in 43% of participants and ≥1 in 85% [34]. The authors reported a statistically significant improvement in OS with a median of 14.9 months with pembrolizumab alone versus 10.7 months with EXTREME for patients with a CPS ≥ 20 [34]. Patients with a CPS ≥ 1 showed a similar benefit with pembrolizumab versus EXTREME, with a median OS of 12.3 versus 10.3 months respectively [34].

A PD-L1 agent, durvalumab, has also been investigated in combination with a CTLA-4 inhibitor, tremelimumab, for R/M HNSCC. The phase III EAGLE trial compared durvalumab with or without tremelimumab to standard-of-care therapy (cetuximab, a taxane, methotrexate, or a fluoropyrimidine) for patients with platinum-refractory R/M HNSCC. Unlike CheckMate 141 and KEYNOTE-040, the trial was negative, failing to demonstrate a statistically significant OS benefit when using durvalumab with or without tremelimumab [46]. Similarly, the KESTREL trial comparing these drugs to the EXTREME regimen for first-line therapy was negative, suggesting a lack of benefit over the standard of care [47].

Following separate positive findings for cetuximab and pembrolizumab in the R/M setting, their combination was tested. Early reports are encouraging and a meta-analysis suggested that the benefit of adding cetuximab to PD-1 inhibition may be greater in HPV-negative than HPV-positive HNSCC, although further prospective studies are necessary to validate these findings [93,94]. One phase II study is testing pembrolizumab plus cetuximab in patients with R/M HNSCC and has thus far demonstrated promising clinical responses regardless of HPV status [93]. A phase III trial “Testing the Addition of Anti-Cancer Drug, Cetuximab, to Standard of Care Treatment (Pembrolizumab) for Returning or Spreading Head and Neck Cancer After Previous Treatment” will help dictate the role of adding EGFR inhibitors to anti-PD-1 agents in HNSCC [95].

The management of R/M nasopharyngeal carcinoma (NPC) is distinct from the management of R/M disease in other HNSCC sites. In NPC, first-line therapy when no surgery or RT option exists is a combination of cisplatin, gemcitabine, and toripalimab [90]. Toripalimab, a PD-1 inhibitor, was FDA-approved for R/M NPC in 2023 based on the multinational JUPITER-02 trial in the first-line setting and POLARIS-02 trial in subsequent-line treatment [37,39]. JUPITER-02, a phase III trial of patients in NPC-endemic regions with treatment-naive R/M NPC, compared cisplatin and gemcitabine +/− toripalimab and found a significantly longer PFS and improved OS in the toripalimab arm [37]. At the four-year follow-up of JUPITER-02, the 5-year OS rates were 52% in the toripalimab arm and 33.9% in the chemotherapy-alone arm [38]. POLARIS-02 then showed an overall response rate (ORR) of 20.5% with a median OS of 17.4 months to toripalimab monotherapy in patients with chemotherapy refractory R/M NPC [39].

National guidelines list other anti-PD-1 agents, tislelizumab and camrelizumab, as first-line therapy in R/M NPC as well. RATIONALE-309 was a phase III trial of treatment-naive patients with R/M NPC who were randomized to gemcitabine and cisplatin with either tislelizumab or placebo. It demonstrated a statistically significant improvement in PFS and a trend toward improved OS in the tislelizumab arm [40]. CAPTAIN-1st was a similar phase III trial that used camrelizumab versus placebo in combination with gemcitabine-cisplatin. The study found a significant improvement in the camrelizumab arm in PFS, OS, and 5-year OS rates [41,42]. Pembrolizumab and nivolumab are listed as options if the others are unavailable [56].

Anti-PD-1 immunotherapy is also offered as a subsequent-line option in the setting of R/M NPC that has progressed after platinum-based therapies, though a key study did not demonstrate an OS benefit [56]. KEYNOTE-122 was a phase III randomized trial of patients with R/M NPC treated with platinum agents that compared second-line or later use of pembrolizumab alone with the investigator’s choice of capecitabine, gemcitabine, or docetaxel. Median OS was not significantly different, though pembrolizumab showed a lower rate of grade 3–5 treatment-related adverse effects [96].

### 4.2. Neoadjuvant and Perioperative Immunotherapy

The rationale for neoadjuvant immunotherapy in resectable HNSCC is grounded in tumor immunobiology. Administering an ICI while the primary tumor remains in situ may more effectively prime and expand tumor-reactive T cells, since more tumor-associated antigens would be available to the immune system compared to after surgical resection [97]. Preclinical models and early clinical data suggest that neoadjuvant immunotherapy may confer greater antitumor efficacy than postoperative administration, potentially through the rapid expansion of tumor-specific CD8+ T cells leading to systemic control of micrometastatic disease [98]. An additional advantage of the neoadjuvant approach is the opportunity to directly evaluate immune remodeling and pathologic tumor regression in the resected specimen [99].

Early-phase studies established the feasibility of the neoadjuvant strategy. In the phase I/II CheckMate-358 neoadjuvant cohort, patients with resectable HNSCC received nivolumab prior to surgery [30]. Although no pathologic complete responses were seen, pathologic tumor regression occurred in 23.5% of HPV-positive tumors and 5.9% of HPV-negative tumors. Although HPV-positive HNSCC generally carries a more favorable prognosis and may exhibit a more immunologically active tumor microenvironment, HPV status alone is not currently used to select patients for PD-1/PD-L1 blockade. Similarly, in a phase II study of neoadjuvant pembrolizumab in patients with HPV-negative disease, no pathologic complete responses were observed, but any pathologic response occurred in 44% of patients [100]. No grade 3–4 adverse events occurred after neoadjuvant pembrolizumab [100]. The data evaluating CTLA-4 inhibitors in the neoadjuvant setting through the IMCISION trial also showed reasonable response rates with no recurrences seen in the best responders [53]. Collectively, these early-phase studies demonstrated the safety and feasibility of neoadjuvant immunotherapy for resectable HNSCC with modest pathologic response rates.

Most recently, the phase III KEYNOTE-689 trial evaluated the perioperative strategy, combining neoadjuvant and adjuvant immunotherapy, using pembrolizumab in patients with newly diagnosed, resectable locally advanced (stage III/IVa) HNSCC [36]. The 714 patients in the KEYNOTE-689 trial were randomized into either the experimental arm or control arm. The control arm received surgery followed by RT with or without cisplatin based on investigator choice and local guidelines (standard therapy). The experimental arm received two cycles of neoadjuvant pembrolizumab and then surgery followed by standard adjuvant therapy concomitantly with three cycles of pembrolizumab and 12 adjuvant cycles of pembrolizumab as well [36]. The experimental arm demonstrated a significant improvement in event-free survival and distant metastasis-free survival, with similar locoregional control when compared with standard therapy [36]. The overall survival endpoint was not statistically significant at the first interim analysis, but longer follow-up analyses are ongoing [101].

Taken together, these early-phase findings and the KEYNOTE-689 trial suggest that initiating checkpoint blockade neoadjuvantly, while tumor antigen is still present, may enhance T-cell priming and generate a more robust antitumor immune response [102]. In June 2025, the FDA approved perioperative pembrolizumab for adults with resectable locally advanced HNSCC whose tumors express PD-L1 with CPS ≥ 1. The FDA-approved regimen consists of neoadjuvant pembrolizumab followed by surgery, pembrolizumab with postoperative RT with or without cisplatin, and subsequent adjuvant pembrolizumab [103]. NCCN guidelines have also incorporated perioperative pembrolizumab into treatment pathways for selected patients with stage III–IVa, PD-L1 CPS ≥ 1 HNSCC [90]. These recommendations are specific to pembrolizumab and should not be generalized to all patients with resectable disease or broadly to perioperative immunotherapy.

Several important questions remain in the curative setting. The optimal duration of therapy is uncertain, with many current trials employing two cycles of preoperative treatment followed by an extended period of postoperative treatment [104,105]. Additionally, current FDA approval and NCCN recommendations for perioperative pembrolizumab are limited to selected patients with PD-L1 CPS ≥ 1 disease. The role of biomarkers (such as PD-L1 expression or other immune signatures such as CTLA-4) in selecting patients most likely to benefit from perioperative immunotherapy remains an area of active investigation [106]. In sum, KEYNOTE-689 establishes a role for perioperative pembrolizumab in selected patients with resectable, locally advanced, PD-L1 CPS ≥ 1 HNSCC, but strategies in earlier-stage disease, longer follow-up, correlation with biomarkers, and overall survival data will be necessary to expand the use of neoadjuvant and perioperative ICI therapy in standard practice.

### 4.3. Immunotherapy in the Adjuvant Setting

In patients with resectable, locally advanced HNSCC, perioperative pembrolizumab is now part of the standard of care. However, if the PD-L1 CPS is <1 or neoadjuvant pembrolizumab was not administered, surgery followed by postoperative RT with or without cisplatin is the standard of care for tumors with intermediate or high-risk pathologic features [90]. Despite aggressive multimodality therapy, recurrence rates remain high; adjuvant immunotherapy strategies have therefore been explored with the goal of improving disease control after definitive surgical treatment [11].

The adjuvant approach following completion of standard therapy aims to eliminate residual disease. It targets micrometastatic tumor cells that may persist after definitive therapy by enhancing immune surveillance [107]. The adjuvant strategy, however, presents theoretical challenges. A study of melanoma highlighted these challenges: after surgical resection, the overall tumor antigen burden is markedly reduced, potentially limiting effective T-cell priming and expansion of tumor-specific immune responses [102].

In the phase III IMvoke010 trial, patients with high-risk locally advanced HNSCC who had completed multimodal definitive therapy without disease progression were randomized to receive either atezolizumab or placebo [45]. IMvoke010 failed to demonstrate a benefit for adjuvant immunotherapy: event-free survival was not significantly improved with atezolizumab compared with placebo, and overall survival was similar between groups. On the other hand, NIVOPOST-OP (GORTEC 2018-01) was a phase III trial in patients with resected locally advanced HNSCC with high-risk pathological features. The trial evaluated the use of nivolumab initiated before the standard-of-care postoperative cisplatin-based chemoradiotherapy, continued concurrently with chemoradiotherapy, and then administered as adjuvant therapy as well. NIVOPOST-OP showed that the addition of nivolumab in this setting significantly improved disease-free survival [31]. Nonetheless, adjuvant nivolumab has not been FDA approved nor is it part of the NCCN guidelines. The IMvoke010 and NIVOPOST-OP trials should be evaluated based on the distinct populations and treatment strategies they examined. IMvoke010 evaluated maintenance atezolizumab after definitive multimodal therapy, while NIVOPOST-OP incorporated nivolumab into adjuvant postoperative chemoradiotherapy with continued treatment afterwards. These findings therefore support further investigation into how the timing, treatment setting, and integration of ICIs with definitive therapy influence outcomes, rather than establishing a general benefit for adjuvant immunotherapy.

Overall, while ICIs have transformed the management of R/M HNSCC and have significantly changed the practice of perioperative treatment of HNSCC, their role in the purely neoadjuvant or adjuvant setting continues to evolve [11].

Despite encouraging results in the neoadjuvant, perioperative, and adjuvant settings, integration of immunotherapy with definitive RT paradigms has proven more complex.

### 4.4. Concurrent Immunotherapy with Definitive RT

Integration of immunotherapy with definitive chemoradiation is supported by a strong biological rationale. RT not only induces tumor cell death, but may also enhance antitumor immune responses by increasing tumor antigen release and promoting antigen presentation [108]. Preclinical studies suggest that RT may synergize with ICIs by generating systemic immune responses that can be amplified through PD-1 inhibition [109]. Early murine models demonstrated improved tumor control with the combination of RT and checkpoint inhibitors, particularly when immunotherapy was administered concurrently with RT [110].

These findings provided the basis for large randomized clinical trials evaluating checkpoint blockade alongside standard chemoradiation in locally advanced HNSCC. However, this strategy has not translated into improved clinical outcomes. The phase III JAVELIN Head and Neck 100 trial demonstrated that adding avelumab concurrently with and following cisplatin-based chemoradiation failed to improve progression-free survival as compared to chemoradiation with placebo, leading to early termination for futility [43]. Similarly, the phase III KEYNOTE-412 trial evaluating pembrolizumab with concurrent chemoradiation and maintenance therapy did not meet its primary endpoint of improved event-free survival, despite a numerical trend favoring the immunotherapy arm [35]. Together, these studies did not establish a statistically significant benefit from adding the aforementioned checkpoint-inhibitor regimens to definitive chemoradiation.

Several explanations have been proposed for these negative results, including treatment-related attenuation of antitumor immune responses from concurrent chemoradiation, radiation-induced disruption of lymphocyte function and the TME, and potential mismatches between the timing of ICIs and the immunologic effects of chemoradiation [54,111]. Consequently, research efforts have increasingly shifted toward alternative integration strategies, including perioperative and adjuvant approaches, and newer RT techniques.

Table 1 summarizes the treatment paradigms for cancer of the oral cavity as an example of HNSCC treatment and Table 2 summarizes the systemic therapies recommended for non-nasopharyngeal HNSCC (Table 1 and Table 2).

## 5. Mechanisms of Resistance to Immunotherapy

Resistance to ICIs can be innate (primary) or develop after a period of initial response (secondary) [112,113]. Primary resistance may result from pre-existing tumor, microenvironmental, and host factors that prevent an effective antitumor immune response. These include low tumor neoantigen burden, inadequate antigen presentation, poor T-cell infiltration or T-cell exclusion, immunosuppressive components in the TME, and pre-existing T-cell dysfunction [112,113]. In contrast, secondary resistance develops after an initial response and may result from tumor adaptation under immune pressure, including increased immunosuppressive signaling, T-cell exhaustion, changes in interferon signaling, and loss of antigen presentation [113].

Resistance mechanisms can also be classified as tumor-intrinsic or tumor-extrinsic. Tumor-intrinsic resistance occurs within the tumor cells and may involve changes in antigen presentation and immune signaling pathways [113]. Higher tumor mutational burden is associated with increased immune recognition and has been associated with improved response to ICIs in some tumor types, while tumors with relatively few neoantigens may be less readily identified by T-cells and may respond less effectively to ICIs [112]. Similarly, mutations in genes involved in the antigen-presenting machinery can confer resistance to ICIs due to decreased cytotoxic T-cell recognition and activation [112]. For example, acquired loss-of-function mutations in Janus kinase 1 or 2 can impair interferon signaling and reduce responsiveness to ICIs, while loss of β2-microglobulin can impair MHC class I antigen presentation [112].

The TME also contributes to ICI resistance. PD-1 is expressed on activated T cells and PD-L1 can be expressed by tumor and immune cells. Binding of PD-1 to PD-L1 inhibits T-cell activation [112]. Persistent exposure to tumor antigens and thus chronic T-cell receptor stimulation can drive progressive CD8+ T-cell exhaustion. Some PD-1+ T-cell populations can maintain a partially exhausted phenotype which can be reinvigorated by PD-1 blockade. However, terminally exhausted populations acquire stable epigenetic alterations that limit their responsiveness to ICIs [112,114,115]. Resistance may also involve increased activity of alternative inhibitory checkpoints. Pathways including LAG-3, TIM-3, and TIGIT may represent compensatory mechanisms of immune escape during PD-1/PD-L1 blockade and provide the rationale for targeting these checkpoints alone or in combination with established ICIs, as discussed above. Increased accumulation of immunosuppressive cell types such as Tregs, and T-cell exclusion from the tumor are other mechanisms of resistance [112]. In one study, tumor-associated macrophages were shown to directly remove anti-PD-1 antibodies from the surface of T cells, providing another potential mechanism of resistance [116].

Finally, memory T cells may play a role in the durability of response to ICIs. Increased numbers of intratumoral memory T cells have been associated with better and more durable responses to PD-1 blockade [117]. Conversely, epigenetic factors that are associated with T-cell exhaustion may limit the persistence of memory T cells and long-term response to ICIs [112,114,115]. Patient factors such as age, medical comorbidities (including autoimmune diseases), medication usage, and performance status also likely contribute to the efficacy of ICIs [113].

## 6. Future Directions and Emerging Applications

### 6.1. Bispecific Antibodies and Bifunctional Agents

Bispecific antibodies simultaneously target two tumor-associated antigens or immune-regulatory pathways. They have the potential to enhance antitumor activity and overcome resistance and are therefore an emerging therapeutic approach in cancer treatment. Several bispecific and bifunctional agents are currently being investigated in HNSCC.

Izalontamab (SI-B001) is a bispecific antibody that targets EGFR and human epidermal growth factor receptor 3 (HER3). In a phase I/Ib dose-escalation and dose-expansion trial, izalontamab was evaluated in 60 patients with locally advanced or metastatic epithelial tumors, including six patients with nasopharyngeal carcinoma and three with HNSCC [118]. The most common treatment-related adverse events (TRAEs) in this study were rash (42%), paronychia (25%), and infusion-related reactions (23%) [118].

Bintrafusp alfa is a bifunctional fusion protein that simultaneously targets PD-L1 and sequesters TGF-β, thus targeting two mechanisms of immune suppression [119]. In a phase I dose-expansion cohort of 32 patients with advanced HNSCC, bintrafusp alfa demonstrated a confirmed objective response rate (ORR) of 13%. Grade 3 TRAEs occurred in 34% of patients, with no grade 4 TRAEs or treatment-related deaths reported [119].

Cadonilimab (AK104) is a bispecific antibody targeting PD-1 and CTLA-4. In a phase II trial of 23 patients with previously treated R/M nasopharyngeal carcinoma, cadonilimab monotherapy produced an ORR of 26.1%, while grade ≥ 3 TRAEs occurred in 8.3% of patients [120,121]. Cadonilimab has also been studied in combination with chemotherapy in patients with anti-PD-1-resistant R/M nasopharyngeal carcinoma. In a phase II study, six of 10 patients achieved an objective response and grade ≥ 3 TRAEs occurred in four of 15 patients (26.7%) [122]. These findings remain preliminary given the small study population and limited follow-up.

Amivantamab is a bispecific antibody targeting EGFR and mesenchymal-epithelial transition factor (MET). In the phase Ib/II OrigAMI-4 study of patients with R/M HNSCC whose disease had progressed following anti-PD-(L)1 therapy and platinum-based chemotherapy, amivantamab demonstrated antitumor activity [123]. In the analysis of the full cohort of 102 patients, the ORR was 42%, including a complete response rate of 15% [124]. Median progression-free survival was 6.8 months, and median overall survival was 12.5 months. These results support continued investigation of dual EGFR/MET targeting as a therapeutic strategy in previously treated R/M HNSCC.

Although these early studies demonstrate the potential of dual targets in oncogenic or immune pathways, most available data are derived from small, early-phase trials, and larger randomized studies are needed to establish the role of bispecific therapies in HNSCC.

### 6.2. Adoptive T-Cell Therapy (CAR-T Cell Therapy)

Chimeric antigen receptor (CAR)-T cell therapy is a type of adoptive cellular immunotherapy in which a patient’s T cells are genetically engineered to recognize and kill cancer cells. The CAR is a synthetic receptor that combines an antibody-derived antigen-binding domain with intracellular signaling domains. This allows T cells to recognize tumor antigens independent of MHCs. CAR-T cell therapy has been successful in treating hematologic malignancies, but results in solid tumors like HNSCC have been more limited. Major challenges include identifying tumor-specific targets, overcoming antigen heterogeneity, improving T-cell trafficking into tumors, and counteracting the immunosuppressive TME [125].

Several tumor-associated antigens have been investigated as potential targets for CAR-T cell therapy in HNSCC. Ideal targets are markers that are highly expressed on tumor cells and minimally expressed on normal tissues. Preclinical data from Haist et al. support CD44v6 as a promising target, with CD44v6-directed CAR-T cells demonstrating selective cytotoxicity against CD44v6-expressing HNSCC cells in vitro [126]. MUC1-targeted CAR-T cells and the addition of a structure that secretes IL-22 have shown promising antitumor activity in HNSCC, improved survivability of the T cells, and increased cytotoxic activity in preclinical models [127]. Additional targets currently being investigated in HNSCC include EGFR, HER2, CD70, and B7-H3 [128,129,130]. Ongoing research focuses on optimizing CAR design and combining CAR-T therapy with other immunotherapies to improve efficacy in solid tumors.

While most CAR-T cell approaches rely on systemic infusion, one study evaluated the feasibility of direct intratumoral CAR-T cell delivery to enhance local efficacy and reduce off-tumor toxicity. A phase I dose-escalation trial in locally advanced/recurrent HNSCC used immunotherapy that was engineered to express a pan-ErbB-targeted CAR and an IL-4-responsive chimeric cytokine receptor [131]. Patients received the autologous T cells as a one-time treatment injected directly into the target lesion(s) without prior lymphodepletion. Manufacturing was successful in all cases; there were no dose-limiting toxicities, and all treatment-related adverse events were grade 2 or less. Disease stabilization at 6 weeks was observed in 9/15 patients (60%). One patient achieved a durable complete response lasting >3 years following subsequent pembrolizumab and talimogene laherparepvec, an oncolytic herpes simplex virus type 1. However, since this patient received multiple additional therapies, the individual contribution of the CAR-T cell therapy cannot be determined [131]. Intratumoral approaches are being investigated in other malignancies, including glioblastoma, where systemic CAR-T cell approaches are limited by barriers to effective tumor trafficking and infiltration [132]. Further investigation is needed to establish the feasibility, efficacy, and potential clinical role of intratumoral CAR-T cell delivery in HNSCC.

CAR-T cell therapy poses a unique set of potentially severe toxicities. Following treatment, patients need to be monitored in the hospital with multidisciplinary intensive management. Cytokine release syndrome (CRS) and immune effector cell-associated neurotoxicity syndrome (ICANS) are two recognized side effects. CRS causes fever, hypotension, and organ dysfunction. ICANS causes confusion, aphasia, seizures, and cerebral edema. Both are managed with supportive care: tocilizumab (IL-6 blockade) for CRS and corticosteroids for ICANS. Additional side effects from these therapies include persistent cytopenias, infection, and damage to healthy tissues that express the target antigen [133].

Another adoptive T-cell therapy that is currently being investigated is TIL therapy. TIL utilizes and improves endogenous antitumor T cells derived directly from a patient’s tumor. While CAR-T cells are peripheral-blood T cells that are engineered to express a synthetic receptor, in TIL, cells are grown ex vivo directly from a solid tumor [134]. Given the association between high TIL density and improved outcomes in HNSCC, this strategy has been explored in the R/M setting following progression on standard therapies [125]. In the phase II C-145-03 trial (NCT03083873), patients with heavily pretreated R/M HNSCC received an autologous TIL infusion following non-myeloablative lymphodepletion and subsequent IL-2 infusion [135]. The objective response rate was 11% (6/53) and all were partial responses [135]. The disease control rate was 76% with a median duration of response of 7.6 months at a median follow-up of 17.9 months [135].

TIL therapy toxicity is mostly associated with the treatments surrounding the therapy: non-myeloablative lymphodepletion chemotherapy and post-infusion high-dose IL-2, rather than TIL therapy itself [136]. The most common toxicities include cytopenias, fever, and gastrointestinal symptoms. Severe adverse events include CRS, capillary leak syndrome, and cardiac dysfunction. Toxicities usually present and resolve within two weeks following TIL therapy, but often require supportive care [136].

Collectively, these studies show both the promise and ongoing challenges of new adoptive T-cell therapies in HNSCC. There is a need for continued research in target selection, treatment delivery mechanisms, and minimizing toxicity.

### 6.3. Therapeutic Vaccines

HPV-positive HNSCC contains the viral E6 and E7 oncoproteins which provide an immunotherapeutic opportunity because they are tumor-associated antigens that can be targeted by therapeutic vaccination. In contrast, HPV-negative HNSCC lacks these distinct viral antigens, and therefore requires an alternative approach such as personalized vaccines that target tumor-specific neoantigens. Therapeutic vaccines aim to generate tumor-specific cellular immunity in HPV-driven cancers to enhance antitumor T-cell responses in patients with HNSCC. Synthetic long-peptide vaccines targeting HPV16, including ISA101 and ISA101b, have been evaluated in early-phase studies of advanced HPV16-positive HNSCC. These vaccines consist of overlapping long peptides derived from HPV16 E6 and E7 oncoproteins designed to stimulate CD4+ and CD8+ T-cell responses. In a single-arm phase II study of 24 patients with incurable HPV16-related cancers, including 22 with oropharyngeal cancer, ISA101 combined with nivolumab produced an ORR of 33% with a median duration of response of 10.3 months [137]. In a subgroup analysis of 22 patients from the previous trial with R/M HPV16-positive oropharyngeal cancer treated with ISA101 plus nivolumab, the ORR was 36%, with two complete responses remaining ongoing after 4.5 years [138].

In a nonrandomized phase I/II trial, 50 patients with advanced HPV-associated cancers received the combination of PDS0101, PDS01ADC, and bintrafusp alfa [139]. PDS01ADC is an IL-12-based immune activator. The ORR was 35.7% (5/14) in ICI-naive patients, with a median OS of 42.4 months, compared with an ORR of 16.7% (6/36) and median OS of 15.8 months in ICI-resistant patients. Among the eight patients with HPV-16-positive tumors who were ICI-naive, the ORR reached 62.5% (5/8). These early results suggest antitumor activity of the combined regimen, particularly in the ICI-naive group, but an unclear benefit for those who are ICI-resistant. However, grade 3 or 4 toxicities occurred in 52% (26/50) of patients [139].

Personalized vaccines, such as TG4050, are also being investigated in HPV-negative HNSCC. TG4050 is an individualized neoantigen vaccine that was evaluated in a randomized phase I trial of patients with resected, locally advanced HPV-negative HNSCC. After a median follow-up of 28.5 months, all of the 16 patients who received TG4050 remained disease-free, compared with 13 of 16 patients in the control arm [140,141]. TG4050 was also well tolerated, with no grade ≥ 3 TRAEs reported [141]. These early findings suggest that personalized neoantigen vaccination may reduce the risk of recurrence, although larger studies are needed to establish clinical efficacy.

DNA-based therapeutic vaccines are another way to enhance HPV-specific immunity through antigen expression and cytokine co-stimulation. MEDI0457, a DNA immunotherapy encoding HPV16/18 E6/E7 antigens with IL-12 plasmids, was administered to 22 patients with locally advanced p16-positive HNSCC in a phase Ib/II study [142]. The regimen was well tolerated, and 18 of 21 evaluable patients demonstrated increased HPV-specific T-cell responses, with durable immune responses persisting to 1 year. Interestingly, one patient who later developed metastatic disease was treated with anti-PD-1 therapy and achieved a rapid and durable complete response, an observation that warrants further investigation but does not establish synergy [142].

Post-treatment therapeutic vaccination strategies are also being explored to augment immune surveillance in patients without active disease. A phase I study of the HPV16 DNA vaccine AMV002 was conducted in 12 patients with previously treated HPV-associated oropharyngeal squamous cell carcinoma and no R/M disease after treatment completion [143]. Post-vaccination, 10/12 patients (83.3%) demonstrated enhanced E6 and/or E7-specific T-cell responses [143]. These early-phase studies suggest that several therapeutic vaccines can induce HPV-related or tumor-specific immune responses, but their clinical efficacy remains under investigation.

### 6.4. Immunotherapy and Proton Beam Therapy

Most clinical trials assessing the combination of immunotherapy and RT for patients with cancer, including lung, breast, and head and neck, utilize photon-based RT [144,145,146]. However, recent studies have provided growing clinical evidence supporting the use of proton beam therapy (PBT) in selected patients with HNSCC. PBT delivers precise RT while reducing exposure to nearby critical structures, such as pharyngeal constrictor muscles, salivary glands, cochlea, brainstem and spinal cord, which provides a dosimetric rationale for reducing side effects [147,148,149]. The first phase III, randomized trial assessing PBT in patients with oropharyngeal cancer has demonstrated non-inferiority for progression-free survival, statistically significant improvement in overall survival, and reduced high-grade toxicity compared to photon therapy [150]. Observational and comparative studies assessing PBT in patients with nasopharyngeal cancer have found either comparable or improved oncologic outcomes and decreased incidences of acute grade 3 toxicity [151,152]. The 2026 American Society for Radiation Oncology (ASTRO) PBT Model Policies recognize head and neck cancer among the clinical settings where PBT may be appropriate [153].

Despite encouraging evidence supporting the use of PBT, the potential benefit of combining PBT with immunotherapy remains uncertain. There are few published studies examining the combination of immunotherapy with PBT specifically, with no completed prospective trials to date. Bo et al. conducted a retrospective, hypothesis-generating study that reported longer durations of response and progression-free survival with proton versus photon RT during ICI treatment [154]. Additional research, including prospective studies, focusing on the combination of immunotherapy and PBT is warranted before a clinical benefit can be established.

## 7. Conclusions

HNSCC tumors utilize many immune-evading pathways to facilitate tumor survival. Key signals in these pathways serve as potential targets for immunotherapy. Certain anti-PD-1 agents have already demonstrated efficacy in HNSCC treatment. ICIs are now an established component of standard-of-care treatment for R/M HNSCC and selected patients with resectable, locally advanced HNSCC in the perioperative setting, while their roles in neoadjuvant and adjuvant settings continue to evolve. However, the efficacy of ICIs can be limited by primary or secondary resistance. CTLA-4 inhibitors have been trialed in HNSCC but insufficient evidence on survival benefit has limited their incorporation into the standard of care. LAG-3, TIGIT, TIM-3, and VISTA also contribute to immune evasion in HNSCC, although their inhibitors have not demonstrated sufficient efficacy in clinical trials. HPV-positive HNSCC is characterized by a more immunogenic TME which could theoretically provide increased susceptibility to immunotherapy. Despite this, its established prognostic advantage is largely confined to oropharyngeal SCC, and HPV positivity has not consistently been predictive of greater sensitivity to immunotherapy. Combining RT with ICIs has thus far yielded negative results in the definitive setting. Still, the advent of PBT opens opportunities for experimentation with the combination of RT and ICIs. Other than ICIs, immunotherapy encompasses methods including CAR-T cell therapy and therapeutic vaccines against oncogenic HPV subtypes that offer additional emerging treatments for HNSCC. Throughout the trials reviewed, immunotherapy has been implemented as monotherapy, concurrently with chemotherapy or RT, or sequentially with these treatment modalities. Future work should examine how treatment sequencing among immunotherapy, chemotherapy, and RT can be optimized. Additionally, many emerging immunotherapeutic strategies and targets are supported primarily by preclinical or early-phase clinical data and therefore warrant further prospective clinical evaluation.

## Figures and Tables

**Table 1:** Treatment paradigm according to NCCN category 2A or above recommendations for cancer of the oral cavity [90]. Immunotherapies are bolded.

Clinical Staging	Treatment of Primary	Adjuvant
T1–2, N0	Surgery: resection of primary +/− neck dissection or sentinel lymph node biopsy	No positive nodes or pathologic features: follow up One positive node, no pathologic features: consider RT Adverse pathologic features: consider re-resection, RT or systemic therapy/RT
	Definitive RT	
T3, N0 or T1–3, N1–3 or T4a, N0–3	Surgery with neoadjuvant pembrolizumab if PD-L1 positive with CPS ≥1 (except for N3 disease)	If neoadjuvant pembrolizumab: RT + pembrolizumab, with cisplatin for positive margins and/or extranodal extension, followed by adjuvant pembrolizumab alone If no adverse pathological features: consider RT Adverse pathological features: systemic therapy/RT, re-resection, or RT
	Clinical trials	
T4b, N0–3 or Unresectable nodal disease or Unfit for surgery	Clinical trial (preferred)	
	PS 0–2: systemic therapy/RT or RT	
	PS 3–4: Palliative RT or single-agent systemic therapy (PS 3 only) or best supportive care	
Metastatic disease at initial presentation	Clinical trial (preferred)	
	PS 0–1: combination systemic therapy, single-agent systemic therapy, surgery, or best supportive care	Clinical trial (preferred), systemic therapy, palliative RT, or best supportive care
	PS 2–3: combination (PS 2 only) or single-agent systemic therapy, best supportive care +/− palliative RT, or palliative surgery	Best supportive care or alternate single-agent systemic therapy or palliative RT
	PS 4: best supportive care +/− palliative RT	

Abbreviations: PS: performance status. For systemic therapy options, which also include immunotherapies, see Table 2.

**Table 2:** Systemic therapies for non-nasopharyngeal cancer according to NCCN category 2A or above recommendations [90]. Immunotherapies are bolded.

Clinical Setting	Regimen	NCCN Category
Definitive concurrent systemic therapy/RT	High-dose cisplatin	1
	Carboplatin + fluorouracil	
	Weekly cisplatin	2A
	Docetaxel, if cisplatin-ineligible	
Induction/sequential therapy	Cisplatin + docetaxel + fluorouracil	1 *
	Cisplatin + fluorouracil + paclitaxel	2A
Neoadjuvant/perioperative immunotherapy	Neoadjuvant pembrolizumab followed by pembrolizumab/RT ± cisplatin and adjuvant pembrolizumab	2A
Postoperative systemic therapy/RT	Cisplatin	1 ^#^
	Docetaxel, if cisplatin-ineligible	2A
Reirradiation + concurrent systemic therapy	Cisplatin + RT	2A
Systemic therapy/RT following induction or for selected recurrent/persistent disease	(Weekly carboplatin or cetuximab) + RT	2A
Recurrent/unresectable/metastatic disease: First line Subsequent line Other combination therapy Single-agent therapy Selected R/M settings	Pembrolizumab + cisplatin or carboplatin + fluorouracil	1
	Pembrolizumab monotherapy for PD-L1 CPS ≥ 1	
	Nivolumab or Pembrolizumab after progression on/after platinum, if not previously used	1
	Cisplatin or carboplatin + fluorouracil + cetuximab, or: Platinum + taxane ± cetuximab	1
		2A
	Platinum + taxane + pembrolizumab	
	Cisplatin + (fluorouracil or cetuximab)	
	Capecitabine, carboplatin, cisplatin, docetaxel, fluorouracil, methotrexate, or paclitaxel	2A
	Cetuximab	
	Cetuximab + nivolumab or pembrolizumab (for SCC)	2A
	Paclitaxel + cetuximab	
	Pembrolizumab for MSI-H, dMMR, or TMB-H tumors	
Selected ethmoid/maxillary sinus cancers	Cisplatin or carboplatin + etoposide	2A

* Category 1 if induction therapy is chosen. ^#^ Category 1 for high-risk non-oropharyngeal cancers. Abbreviations: SCC: squamous cell carcinoma; MSI-H: microsatellite instability-high; dMMR: deficient mismatch repair; TMB-H: tumor mutational burden-high.

## Data Availability

No new data were created or analyzed in this study. Data sharing is not applicable to this article.

## Abbreviations

The following abbreviations are used in this manuscript:

HNC	Head and neck cancer
HNSCC	Head and neck squamous cell carcinoma
HPV	Human papillomavirus
EBV	Epstein–Barr virus
OS	Overall survival
RT	Radiation therapy
Tregs	Regulatory T cells
TME	Tumor microenvironment
NK	Natural killer
PD-1	Programmed cell death protein-1
PD-L1	Programmed death ligand-1
PD-L2	Programmed death ligand-2
APC	Antigen-presenting cell
MHC	Major histocompatibility complex
ITIM	Intracellular immunoreceptor tyrosine-based inhibitory motif
ITSM	Immunoreceptor tyrosine-based switch motif
SHP-1/2	Src homology region 2 domain-containing phosphatases 1/2
IFN-γ	Interferon-γ
JAK2	Janus kinase 2
NF-κB	Nuclear factor kappa-light-chain-enhancer of activated B cells
HIF	Hypoxia-inducible factor
R/M	Recurrent/metastatic
EGFR	Epidermal growth factor receptor
CTLA-4	Cytotoxic T Lymphocyte Antigen 4
TGF-β	Transforming growth factor-β
LAG-3	Lymphocyte activation gene-3
TIL	Tumor-infiltrating lymphocyte
TIGIT	T-cell immunoreceptor with immunoglobulin and immunoreceptor tyrosine-based inhibitory motif domains
TIM-3	T-cell immunoglobulin mucin-3
VISTA	V-domain Ig suppressor of T-cell activation
PFS	Progression-free survival
CPS	Combined positive score
SCC	Squamous cell carcinoma
MSI-H	Microsatellite instability-high
dMMR	Deficient mismatch repair
TMB-H	Tumor mutational burden-high
NPC	Nasopharyngeal carcinoma
ICI	Immune checkpoint inhibitor
CAR	Chimeric antigen receptor
CRS	Cytokine release syndrome
ICANS	Immune effector cell-associated neurotoxicity syndrome
PBT	Proton beam therapy
ASTRO	American Society for Radiation Oncology
HER3	Human Epidermal Growth Factor Receptor 3
